# Evaluation of image quality at the detector’s edge of dedicated breast positron emission tomography

**DOI:** 10.1186/s40658-020-00351-6

**Published:** 2021-01-18

**Authors:** Yoko Satoh, Utaroh Motosugi, Masamichi Imai, Yoshie Omiya, Hiroshi Onishi

**Affiliations:** 1 Yamanashi PET Imaging Clinic, Shimokato 3046-2, Chuo City, Yamanashi Prefecture 409-3821 Japan; 2 Department of Radiology, University of Yamanashi, Chuo City, Yamanashi Prefecture Japan; 3 Department of Radiology, Kofu-kyoritsu Hospital, Kofu City, Yamanashi Prefecture Japan

**Keywords:** Dedicated breast positron emission tomography, ^18^F-Fluorodeoxyglucose, Image quality, Edge of detector

## Abstract

**Background:**

Using phantoms and clinical studies in prone hanging breast imaging, we assessed the image quality of a commercially available dedicated breast PET (dbPET) at the detector’s edge, where mammary glands near the chest wall are located. These are compared to supine PET/CT breast images of the same clinical subjects.

**Methods:**

A breast phantom with four spheres (16-, 10-, 7.5-, and 5-mm diameter) was filled with ^18^F-fluorodeoxyglucose solution (sphere-to-background activity concentration ratio, 8:1). The spheres occupied five different positions from the top edge to the centre of the detector and were scanned for 5 min in each position. Reconstructed images were visually evaluated, and the contrast-to-noise ratio (CNR), contrast recovery coefficient (CRC) for all spheres, and coefficient of variation of the background (CV_B_) were calculated. Subsequently, clinical images obtained with standard supine PET/CT and prone dbPET were retrospectively analysed. Tumour-to-background ratios (TBRs) between breast cancer near the chest wall (close to the detector’s edge; peripheral group) and at other locations (non-peripheral group) were compared. The TBR of each lesion was compared between dbPET and PET/CT.

**Results:**

Closer to the detector’s edge, the CNR and CRC of all spheres decreased while the CV_B_ increased in the phantom study. The disadvantages of this placement were visually confirmed. Regarding clinical images, TBR of dbPET was significantly higher than that of PET/CT in both the peripheral (12.38 ± 6.41 vs 6.73 ± 3.5, *p* = 0.0006) and non-peripheral (12.44 ± 5.94 vs 7.71 ± 7.1, *p* = 0.0183) groups. There was no significant difference in TBR of dbPET between the peripheral and non-peripheral groups.

**Conclusion:**

The phantom study revealed poorer image quality at < 2-cm distance from the detector’s edge than at other more central parts. In clinical studies, however, the visibility of breast lesions with dbPET was the same regardless of the lesion position, and it was higher than that in PET/CT. dbPET has a great potential for detecting breast lesions near the chest wall if they are at least 2 cm from the edge of the FOV, even in young women with small breasts.

## Background

^18^F-Fluorodeoxyglucose (FDG) positron emission tomography/computed tomography (PET/CT) has become one of the most useful tools in diagnostic imaging for cancer. Many studies have demonstrated the efficacy of whole-body FDG-PET/CT in staging or re-staging, in monitoring the response to therapy, and for predicting the prognosis of patients with breast cancer [1–3]. It is important to detect breast cancer at an early stage when the lesions are small, since mortality increases with tumours exceeding 1 cm in size [4, 5]. However, detection of small breast cancers by whole-body PET/CT is challenging because of its limited spatial resolution [6]. High-resolution dedicated breast PET (dbPET) scanners have been developed to detect small breast lesions. There are the two dominant types of high-resolution dbPET, i.e. positron emission mammography (PEM) and a tomographic technique using a ring-shaped scanner (ring-shaped dbPET) [7]. PEM systems depict breast tissue via soft compression of the breast with two opposing plate-like detectors and have higher sensitivity for detecting small lesions than whole-body PET/CT [8–10]. Ring-shaped dbPET scanners do not employ any breast compression and can visualise breast cancer more clearly than whole-body PET/CT [11, 12]. These high-resolution breast PET systems have greater photon sensitivity and can improve spatial resolution by setting the detector close to the breast, reducing respiratory movement (either by fixing the breast to the PEM detector or by scanning in the prone position for dbPET), and using smaller detection units than those of whole-body PET/CT. Their performances have been evaluated using NEMA-NU4-2008 standards [13], and the physical parameters of dbPET and whole-body PET/CT have been compared using a common breast phantom [14]. In that comparative study, the breast phantom was located at the centre of each scanner, and no studies have reported on the quality of dbPET images close to the edge of the detector. However, many Japanese women have small breasts, and their mammary glands are often located near the chest wall, close to the edge of detector, even when they are in the prone position. This tendency is particularly common in young women who are less likely to have breast ptosis than older women. Therefore, it is necessary to evaluate the consequences of a shift in the position of the breast phantom away from the centre of the detector. This study aimed to confirm the image quality of dbPET at the edge of the detector by phantom and clinical studies and to compare them with clinical PET/CT.

## Methods

This single-institution study was approved by the Institutional Review Board of the Kofu Neurosurgical Hospital and Yamanashi PET imaging clinic in accordance with the Declaration of Helsinki. Because of the retrospective study design and the use of anonymised patient data, the requirement for informed consent was waived.

### Ring-shaped dbPET scanner

The dbPET scanner (Elmammo, Shimadzu Corp., Kyoto, Japan) has received approval from the Japanese Pharmaceutical Affairs Law and is commercially available in Japan. It consists of 36 detector modules arranged in three contiguous rings, has a diameter of 195 mm and a transaxial length of 156.5 mm, and has depth-of-interaction measurement capability [15]. The transaxial effective field-of-view (FOV) is 185 mm. Each detector block consists of a four-layered 32 × 32 array of lutetium oxyorthosilicate crystals (1.44 mm × 1.44 mm × 18 mm in size with each depth-of-interaction (DOI) layer being 4.5 mm tall) coupled to a 64-channel position-sensitive photomultiplier tube via a light guide. Attenuation correction was calculated using a uniform attenuation map with object boundaries obtained from emission data [16]. Scatter correction was performed using the convolution-subtraction method with kernels obtained by background tail fitting [17]. Performance metrics included 1.5-mm FWHM resolution in standard mode in the transverse, sagittal, and coronal views, detector sensitivity of 0.09–0.13 cps/Bq at the centre of the detector, and the sensitivity at 39.5 mm from the edge of the detector (depth of 1/4) is 0.05–0.08 cps/Bq. The peak noise equivalent count (NEC) was 600–800 kcps. The sensitivity values and peak NEC are based on the manufacturer's product specifications, which were not measured in this study; therefore, they show slight variations. The characteristics and standard performance of this scanner have been reported in detail previously [13].

### Whole-body PET/CT scanner

PET/CT scans were obtained using a Biograph Horizon TrueV FDG-PET/CT system (Siemens Medical Solutions, Knoxville, TN, USA). This system has 52 detector rings consisting of 160 blocks, with each block containing an array of 13 × 13 lutetium oxyorthosilicate crystals (4 mm × 4 mm × 20 mm) covering an axial FOV of 221 mm and a transaxial FOV of 690 mm diameter. A CT scan was performed for attenuation correction (130 kV; 15–70 mA; tube rotation time, 0.6 s per rotation; pitch, 1; a transaxial FOV, 700 mm; and section thickness, 5 mm).

### Development and preparation of the breast phantom

A cylindrical breast phantom containing four plastic spheres of different diameters was used. The inner diameter and hight of the cylinder were 100 mm and 140 mm, respectively. The diameters of the spheres, arranged in a planar circle inside the phantom, were 5, 7.5, 10, and 16 mm. Spheres smaller than 5 mm in diameter were not used because they could not be detected by PET/CT. Furthermore, in our previous studies with low TBR phantoms, the smallest 5-mm-diameter sphere could not be visually detected on dbPET images when the sphere-to-background activity concentration was less than 8:1 [14]. Therefore, the visibility of lesions smaller than 5 mm was not evaluated in this study. The cylinder and four spheres were filled with 18F-FDG solution at a sphere-to-background radioactivity concentration ratio of 8:1 in accordance with a previous study [14]. The background radioactivity at the start of data acquisition by dbPET was set to 2.46 kBq/mL. One scan was performed under each position as detailed in the next section.

### Data acquisition and image reconstruction

**Fig. 1:**
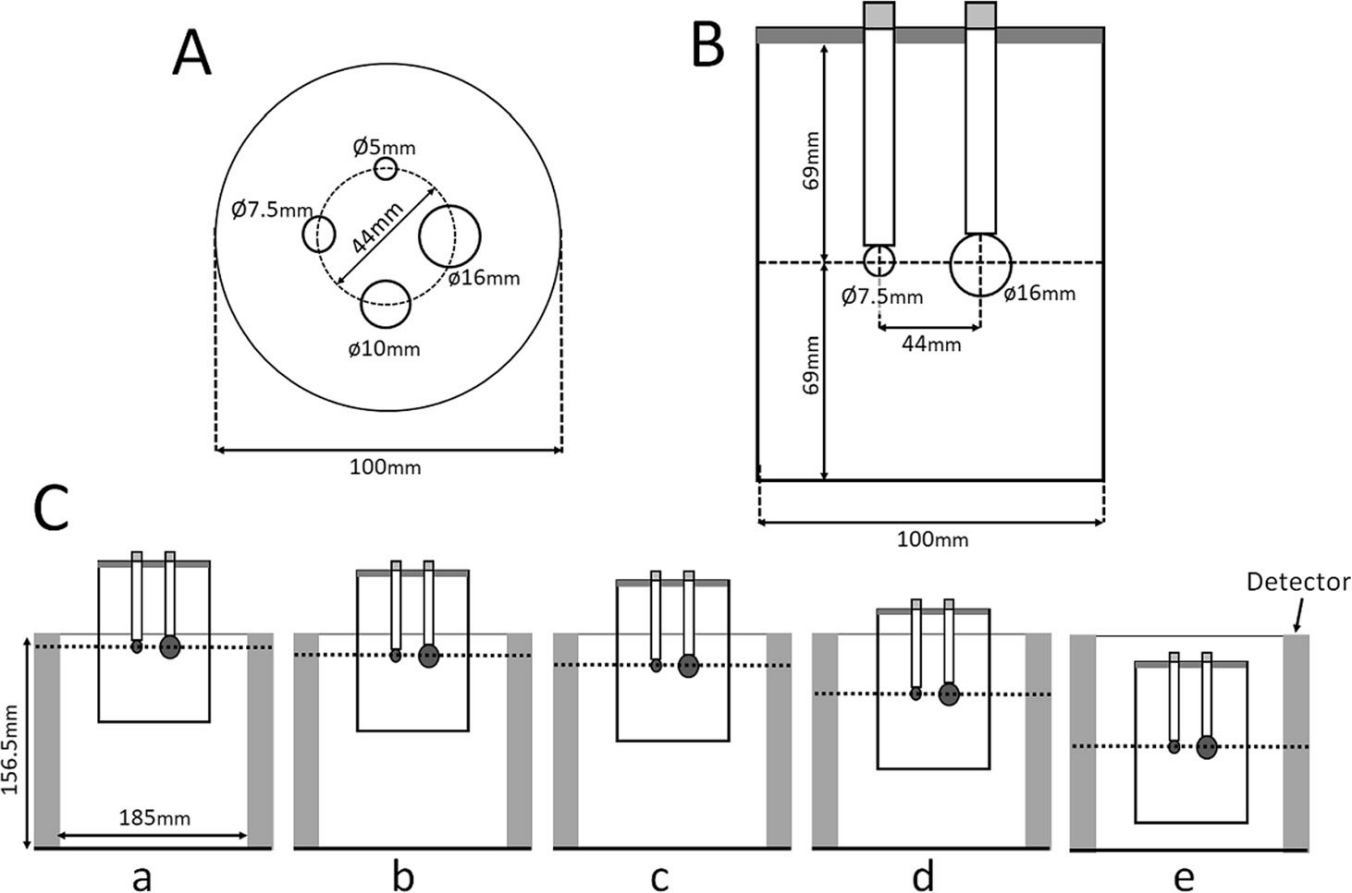
Cross-sections of the phantom with dimensions. A transverse plane view (**A**) and a lateral view (**B**) of the breast phantom in which the spheres were arranged. **C** Relationship between the PET detectors and the phantom (lateral views). The distances from the upper edge of the detector were 8 mm (a), 13 mm (b), 19.5 mm (c, depth of 1/8), 39.5 mm (d, depth of 1/4), and 78 mm (e, depth of 1/2, the centre of the detector)

The breast phantom was positioned such that the spheres were precisely located in the same transverse plane at different positions in the transverse field of view. The spheres were positioned at 8 mm, 13 mm, 19.5 mm (1/8 of detector axial FOV), 39 mm (1/4 of detector axial FOV), and 78 mm (1/2 or halfway point of the detector axial FOV) below the top edge of the detector (Fig. 1). Since it is unlikely that a breast lesion is located at the bottom edge of the detector, only the chest wall side of the detector was evaluated. Sphere placement at each position in the detector was confirmed visually and by measurement on the image. A three-dimensional list-mode dynamic row-action maximum-likelihood algorithm (LM-DRAMA) was applied for the reconstruction of a dbPET image. DRAMA has been shown to achieve fast converge with a reasonable signal-to-noise ratio with a single iteration and 128 subsets by including the relaxation parameter *λ*, which was defined by the subset number and the relaxation control parameter of β [18, 19]. In this study, the dbPET images were reconstructed using LM-DRAMA with β = 20, the matrix size in the transverse view 236 × 200 × 236, and a post-reconstruction smoothing Gaussian filter (1.17-mm FWHM) without the time-of-flight (TOF) algorithm according to the previous report [14]. For the clinical images, the extracted contour was the same as the subject's boundary and was therefore used for the attenuation coefficient map without adjustment. For the phantom images, the estimated contour of the boundary was adjusted to account for the wall thickness of the phantom. The reconstructed voxel size of the dbPET images was 0.78 mm × 0.78 mm × 0.78 mm.

The clinical PET/CT images were reconstructed using the ordered subset expectation maximisation method and the TOF algorithm with four iterations and 10 subsets. The CT data were resized from a 512 × 512 matrix to a 180 × 180 matrix to match the PET data and construct CT-based transmission maps for attenuation correction of the PET data with a post-reconstruction smoothing Gaussian filter (5 mm FWHM). The reconstructed voxel size of the PET/CT images was 4.11 mm × 4.11 mm × 5 mm.

The volume per voxel of the dbPET image is 0.47 mm^3^, while that of the PET/CT image is 84.5 mm^3^, which is 178 times larger than that of the dbPET.

### Analyses of phantom image quality

Visual and quantitative analysis of all PET images was performed using an imaging workstation equipped with syngo.via VB10 software (Siemens Healthcare GmbH, Erlangen, Germany). Standardised uptake values (SUVs), as a semi-quantitative assessment of FDG accumulation, were extracted using this software. The SUV of a given tissue was calculated using the following formula:

$$ \mathrm{SUV}=\frac{\mathrm{Tumour}\ \mathrm{activity}\ \mathrm{concentration}\ \left(\mathrm{Bq}/\mathrm{ml}\right)}{\mathrm{Injected}\ \mathrm{dose}\ \left(\mathrm{Bq}\right)}\kern0.5em \times \mathrm{body}\ \mathrm{weight}\ \left(\mathrm{g}\right) $$

The maximum (SUV_max_) and the mean (SUV_mean_) SUVs are the maximum and average value within the region of interest (ROI) (or volume of interest [VOI]), respectively.

An experienced nuclear medicine physician and two experienced PET technologists evaluated the hot spheres. Evaluations were performed using the slices displayed in the coronal image slice containing the sphere centres. The 5-mm-diameter hot sphere was visually graded as follows: 2, identifiable; 1, visualised, but similar hot spots observed elsewhere; and 0, not visualised. Spheres with visual scores of ≥ 1.5 were deemed to be detectable. The final score for the visibility of the smallest sphere was the mean of the scores from three readers. The visual assessment was performed based on the Japanese guidelines [20]. A circular ROI with a diameter of 5 mm was placed on the central slice of the 5-mm hot sphere. Additionally, 12 ROIs with a diameter of 5 mm were placed in the background region of the coronal image slice that contained the sphere centres, and 12 ROIs were placed in the + 5-mm and – 5-mm adjacent slices (36 ROIs in total). The contrast-to-noise ratio (CNR) and contrast recovery coefficient (CRC) were calculated to quantitatively compare the visibility between the different positions in the dbPET detector. CNR and CRC provide information about the visibility and how accurately the system reproduces the true activity concentration, respectively. A modified CNR was calculated as follows:

$$ \mathrm{CNR}=\frac{\left|{C}_{\mathrm{H}}-\overline{C_{\mathrm{B},5\mathrm{mm}}}\right|}{{\mathrm{SD}}_{\mathrm{B},5\mathrm{mm}}}, $$

where *C*_H_ is the SUV_mean_ in each sphere ROI, $\overline{C_{\mathrm{B},5\mathrm{mm}}}$ is the average SUV_mean_ of the background ROIs, and SD_B_,_5mm_ is the standard deviation of the background ROIs.

A modified CRC was calculated as follows:

$$ \mathrm{CRC}=\frac{\left(\raisebox{1ex}{${C}_{\mathrm{H}}$}\!\left/ \!\raisebox{-1ex}{$\overline{C_{\mathrm{B},5\mathrm{mm}}}$}\right.\right)-1}{\left(\raisebox{1ex}{${a}_{\mathrm{H}}$}\!\left/ \!\raisebox{-1ex}{${a}_{\mathrm{B}}$}\right.\right)-1}\times 100\left[\%\right], $$

where *a*_H_ and *a*_B_ are the activity concentration in the hot sphere and the background, respectively.

We also placed 10 ROIs with a diameter of 16 mm in the background region of the coronal image slice that contained the sphere centres and its + 5-mm and – 5-mm adjacent slices (30 ROIs in total).

The modified coefficient of variation (CV_B_) was calculated using the data from these 16 mm ROIs as follows:

$$ \mathrm{CVB}=\frac{{\mathrm{SD}}_{\mathrm{B},16\mathrm{mm}}}{\overline{C_{\mathrm{B},16\mathrm{mm}}}}\times 100\left[\%\right] $$,

where SD_B_,_16mm_ is the standard deviation in the background ROIs and $\overline{C_{\mathrm{B},16\mathrm{mm}}}$ is the average SUV_mean_ of the background ROIs.

These physical values were calculated according to previous reports [14, 21].

### Analysis of human images

Of a total of 202 consecutive women who underwent both dbPET and whole-body PET/CT scans from August 2016 to September 2019, 62 histologically proven breast cancer tumours of 57 women with positive findings on both dbPET and whole-body PET/CT images were included in the study. Patients fasted at least 6 h prior to administration of 18F-FDG (3 MBq/kg) and were scanned at 60 min post-injection by whole-body PET/CT in the supine position for 90 s per bed position. Then, they were scanned at 90 min post-injection by dbPET in the prone position for 7 min per breast. The PET/CT and dbPET images were reconstructed using the same conditions as for the phantom images.

All PET images were evaluated separately by two experienced nuclear medicine physicians (with 16 and 7 years of experience in interpreting PET, respectively). Of the 62 lesions, those in which the shortest distance from the detector edge on the chest wall side to the tumour centre was 2 cm or less on the transverse image of dbPET were defined as the “peripheral group”; the other lesions were defined as the “non-peripheral group”. Non-mass uptakes, other than focus and mass-like uptakes, were excluded because their quantitative reliability could not be established. Tumours that were exactly centred in both peripheral and non-peripheral regions and whose volume was equally present in both regions were also excluded.

The quantitative value of PET is known to be affected by the partial volume effect [22]. To account for lesion size bias, lesion sizes were matched in the peripheral and non-peripheral groups. The non-peripheral group was reorganised such that lesion size matched the peripheral group in a one-to-one correspondence. As a result, 23 lesions in each group (total 46 lesions) were included in the final analysis.

**Fig. 2:**
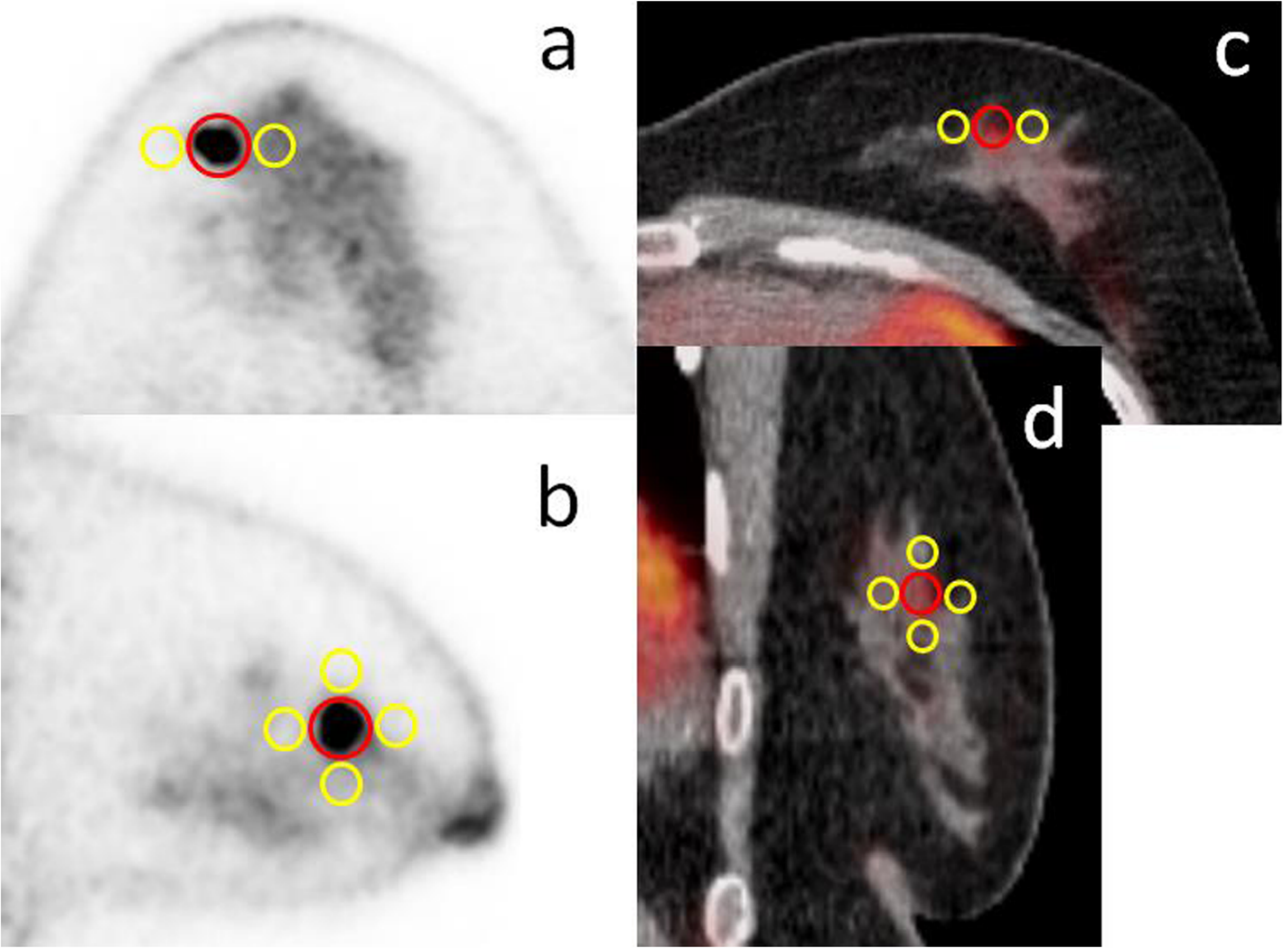
Positioning of the volume of interest (VOI) for the measurement of clinical PET images. A spherical VOI (red) on FDG uptake of the tumour and 5 (or 6) spherical VOIs (yellow) were placed to calculate the TBR. Transaxial and sagittal images of dbPET (**a**, **b**) and whole-body PET/CT (**c**, **d**)

To evaluate lesion visibility in dbPET depending on the position of the tumour, tumour-to-background ratio (TBR) was calculated as follows. All PET images were displayed in an inverse grey scale with a standardised uptake range of 0–6 for the purpose of reducing intra-reader variability. First, the smallest spheroid VOI that just contained the tumour was placed on the monitor. Second, 5-mm-diameter spherical VOIs were placed at 6 locations on the top, bottom, left, right, anterior, and posterior of the tumour, as close as possible to it, in the non-peripheral group. Five VOIs were used in the peripheral group; the posterior VOI was excluded because there was not enough space to place it posterior of the tumour (Fig. 2). The TBR was the SUV_max_ of the VOI on the tumour divided by the average SUV_mean_ of the five or six VOIs on the background.

$$ \mathrm{TBR}=\frac{{\mathrm{SUV}}_{\max \_\mathrm{T}}}{\overline{{\mathrm{SUV}}_{\mathrm{mean}\_\mathrm{B}}}} $$,

where SUV_max_T_ is the SUV_max_ in the tumour and $\overline{{\mathrm{SUV}}_{\mathrm{mean}\_\mathrm{B}}}$ is the average SUV_mean_ of the background VOIs.

In PET/CT, the SUV_max_ and the SUV_mean_ of the same ROI are equal because a 5-mm-diameter spherical VOI contains only one voxel. The TBRs were compared between dbPET and PET/CT images, and the TBR of dbPET was compared between the peripheral and non-peripheral groups.

### Statistical analysis

A paired *t* test was used to compare the TBR of dbPET and whole-body PET/CT for the peripheral and non-peripheral groups, respectively. The Mann–Whitney *U* test was used to test for differences in TBR on dbPET between peripheral and non-peripheral lesion groups. Statistical significance was defined as *p* < 0.05. Additionally, for these PET measurements, interclass correlation coefficients (ICC) were used to evaluate the reliability between readers.

## Results

### dbPET phantom studies

**Fig. 3:**
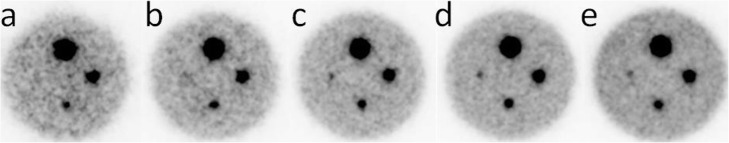
Images of the breast phantom scanned by dbPET at the five different positions. Images **a**, **b**, **c**, **d**, and **e** correspond to phantom images scanned at 8 mm, 13 mm, 19.5 mm (depth of 1/8), 39 mm (depth of 1/4), and 78 mm (depth of 1/2, the centre of the detector) below the top edge of the detector. Closer to the edge of the detector, the background was noisier. The hot spots of the smallest sphere with a diameter of 5 mm could not be confirmed in **a** and **b**

**Fig. 4:**
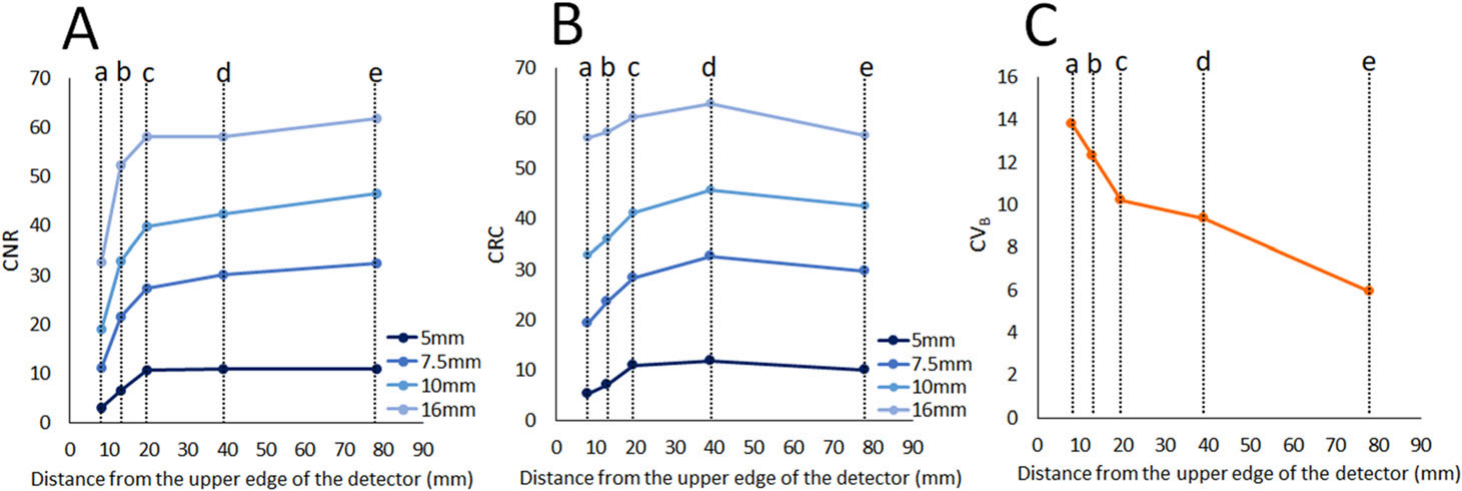
Quantitative assessment of the breast phantom scanned by dbPET at the five different positions. Line graphs of CNR (**A**), and CRC (**B**), and CV_B_ (**C**) of the breast phantom images for the 5 mm, 7.5 mm, 10 mm, and 1 mm spheres scanned by dbPET at the five different positions are shown. The a, b, c, d, and e on the *X*-axis correspond to phantom positions of 8 mm, 13 mm, 19.5 mm (depth of 1/8), 39 mm (depth of 1/4), and 78 mm (depth of 1/2, the centre of the detector) below the top edge of the detector

Images of the breast phantom scanned by dbPET at the five different positions are shown in Fig. 3. In the qualitative evaluation, the visual scores recorded by a nuclear medicine physician and two nuclear medicine technologists on the dbPET images at 8 mm, 13 mm, 19.5 mm (depth of 1/8), 39 mm (depth of 1/4), and 78 mm (depth of 1/2, the centre of the detector) below the top edge of the detector were 0, 0.33, 1.67, 2, and 2, respectively. All other spheres had visual scores of 2. Second, in the quantitative evaluations, the CNR, CRC, and CV_B_ for the 5-mm sphere at the centre of the detector were 10.96, 10.02, and 5.91, respectively (Fig. 4). The CNR and CRC decreased and the CV_B_ increased when the phantom was placed closer to the detector’s edge. Image degradation closer to the edge of the detector was confirmed by visual scoring. Based on the results of this phantom study, the boundary line between peripheral and non-peripheral lesions in clinical studies was defined as 2 cm from the upper edge of the detector.

### Patient studies

A total of 46 lesions (23 in each group) in 45 breasts of 44 patients (age range 37–87 years, mean 57.8 years) were evaluated. One patient had one peripheral and one non-peripheral lesion on one side of her breast, one patient had two peripheral lesions on one side of the breast, and each of the 42 patients had one lesion.

**Fig. 5:**
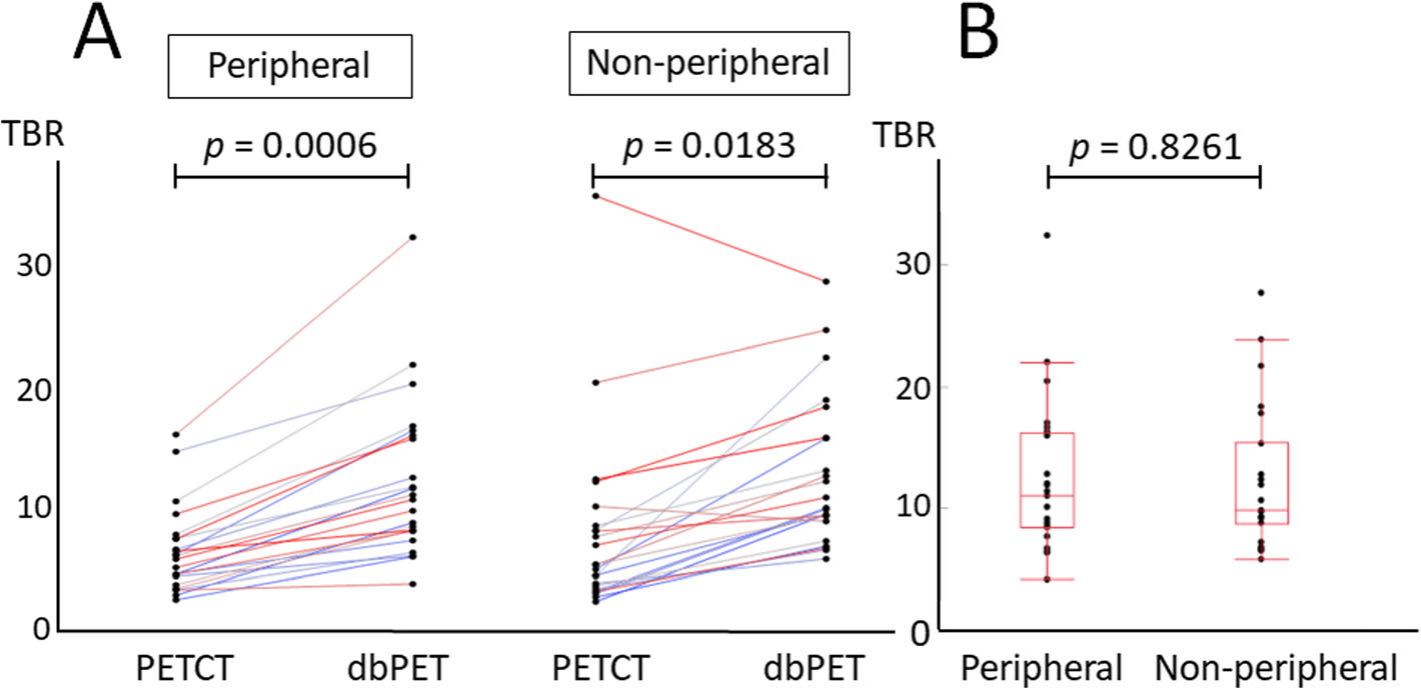
Comparisons of tumour-to-background ratios (TBRs). **a** There were significant differences between TBRs of dbPET and whole-body PET/CT in both peripheral (12.38 ± 6.41 vs 6.73 ± 3.5, *p =* 0.0006) and non-peripheral (12.44 ± 5.94 vs 7.71 ± 7.1, *p =* 0.0183) groups. **b** There was no significant difference in the TBR of dbPET between the peripheral and non-peripheral groups (*p =* 0.8261)

**Fig. 6:**
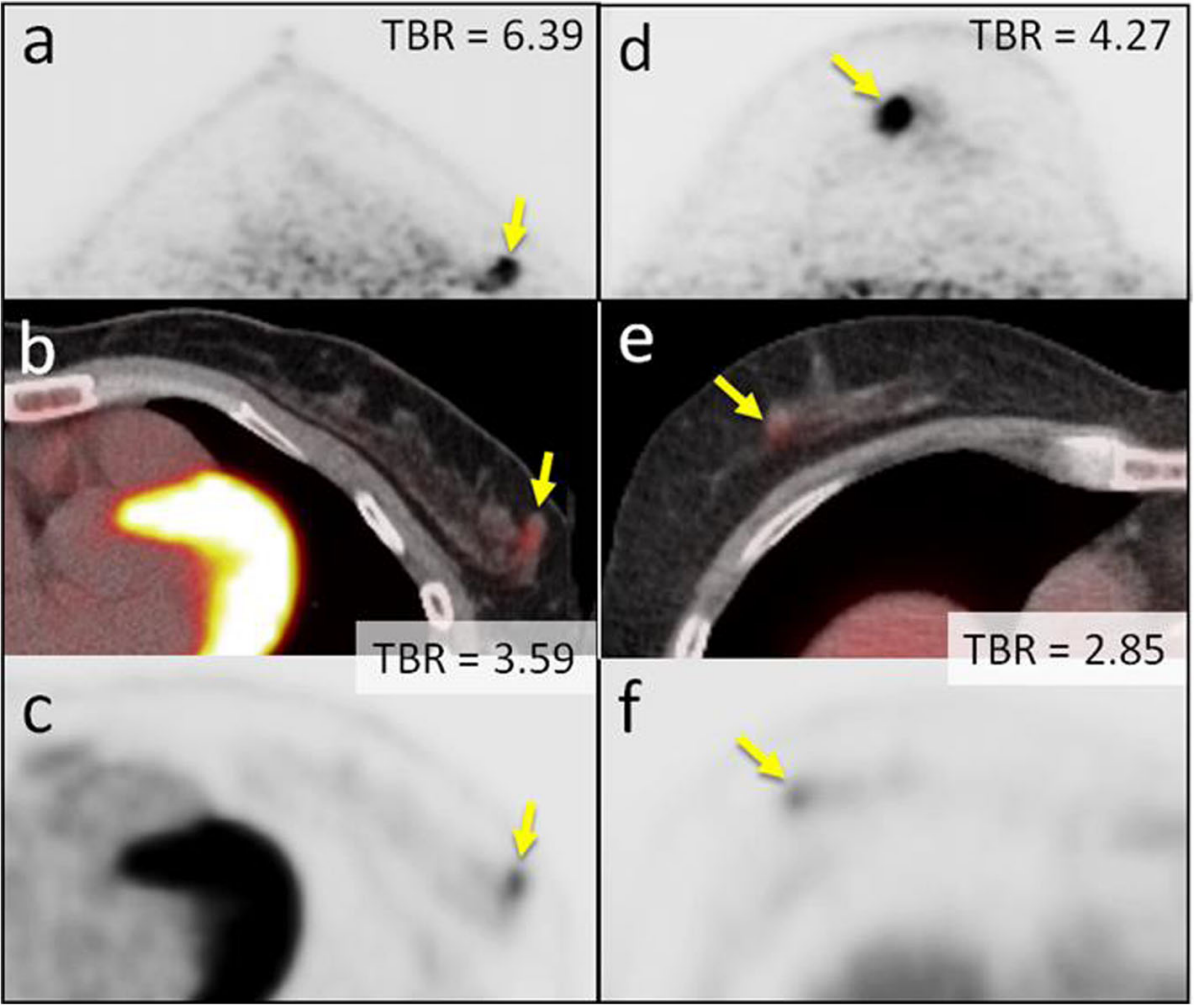
Peripheral and non-peripheral breast cancer images of dbPET scanned in the prone position and whole-body PET/CT scanned in the supine position. Representative clinical images of peripheral (39-year-old woman; clinical size, 12 mm; **a**–**c**) and non-peripheral (63-year-old woman; clinical size, 9 mm; **d**–**f**) breast cancers on dbPET (**a**, **d**), PET/CT (**b**, **e**), and PET (**c**, **f**) with focal FDG uptake in the background mammary gland tissue. Although the focal uptakes were visualised on both the dbPET and whole-body PET/CT images, it was obvious on dbPET with a higher TBR than that on whole-body PET/CT, regardless of the peripheral or non-peripheral location

After propensity score matching for lesion size, the mean diameters of the lesions in the peripheral and non-peripheral groups were 19.3 ± 12 mm and 20 ± 12.2 mm (*p* = 0.7663), respectively (Table 1). The ICC of the TBR was excellent (0.92 for PET/CT and 0.89 for dbPET). The average values evaluated by two readers were analysed in this study. The TBR of dbPET was significantly higher than that of whole-body PET/CT in both the peripheral (12.38 ± 6.41 vs 6.73 ± 3.5, *p* = 0.0006) and non-peripheral groups (12.44 ± 5.94 vs 7.71 ± 7.1, *p* = 0.0183) (Fig. 5a). There was no significant difference in the TBRs of dbPET between the peripheral and non-peripheral (*p* = 0.8261, Fig. 5b). Figure 6 shows representative cases of peripheral and non-peripheral breast cancer acquired by dbPET in the prone position and PET/CT in the supine position. The breast cancers were visualised on dbPET more easily than on PET/CT regardless of the location of the lesion (peripheral or non-peripheral).

**Table 1:** Characteristics of the 46 lesions in 44 patients

Group		Peripheral	Non-peripheral
Number of lesions (women)		23 (22)	23 (23)
Age (years) [median, (range)]		52 (37–87)	62 (43–79)
Clinical size (mm) [median, (range)]		17 (7–51)	17 (7–52)
Distance from chest wall to lesion (mm) [median, (range)]^a^		0.83 (0.44–1.55)	32.7 (20.2–64.7)
Histopathology	Invasive ductal carcinoma	19	20
	Invasive lobular carcinoma	1	0
	Invasive ductal and lobular carcinoma	1	0
	Other invasive carcinomas	1	0
	Ductal carcinoma in situ	1	3
Subtype	Luminal A/B	9/7	10/5
	HER2 positive	1	2
	Triple negative	2	3
	Unknown	4	3

^a^Distance from the FOV margin on the chest wall to the centre of the lesion

## Discussion

In this study, we evaluated the image quality obtained at different coronal locations within the dbPET detector for ring-type breast PET. In the phantom study, the CNRs and CRCs were lower and the CV_Bs_ were higher for all sphere sizes closer to the breast-insert side of the detector. These results indicate that the quantitative image quality was degraded at about 2 cm from the breast-insert or chest wall side of the detector.

Minoura et al. reported that dbPET images show high levels of noise at the edge of the detector (the top of the detector or the chest wall side) and showed the relationship between the slice position in the dbPET image and the standard deviation of noise [23]. Our results showing that dbPET image quality decreases at 19.5 mm from the detector edge are consistent with their reports. The geometric efficiency by Monte Carlo simulation at this depth was 0.2, which was considerably lower than that at the centre, which was 0.65. Usually, whole-body PET scans use overlapping acquisition beds to correct for reduced sensitivity at the detector edges; acquisition of data in overlapped regions can improve quantitative accuracy [24, 25]. However, since the dbPET scanner is fixed and cannot use overlapping acquisition to improve image quality near the edges of the detector, there are concerns that important information may be missed. Additionally, out-of-FOV radioactivity, among which myocardial uptake may be most significant, would also significantly affect image quality. However, the effect of out-of-FOV radioactivity on the dbPET image quality could not be evaluated in this phantom study because such structures that showed high FDG uptake, such as the myocardium, were not included in our phantom. Therefore, to better reproduce the same scatter, singles, and random conditions as in real patients, evaluation with a phantom that simulates out-of-FOV radioactivity from the patients’ chest is necessary, which may detrimentally affect these results.

Based on the phantom test results, the lesion visibility of clinical dbPET images was compared for peripheral lesions located up to 2 cm from the upper edges of the detector and for the other non-peripheral lesions, which revealed that CNR and CRC decreased and CV_B_ increased as the lesions were positioned closer to the 2 cm edge of the dbPET scanner FOV. While in the clinical study, there were significant differences between PET/CT and dbPET TBRs in both groups. There were no significant differences between the peripheral and non-peripheral groups for dbPET. Peripheral and non-peripheral groups could not be compared in whole-body PET/CT, since the patients were scanned in the supine position, with their breasts naturally slumped against their chest walls. The clinical dbPET images had a high TBR in some projection directions, which may have facilitated the detection of lesions. This may be because the phantom image had a uniform background, whereas human breasts have different proportions of mammary glands and fat, and therefore, the physiological FDG uptake in the background tissue was not uniform [26, 27]. Additionally, the TBRs in both dbPET groups were significantly higher than that in PET/CT. dbPET is a higher-resolution scanner than conventional whole-body PET/CT, and the prone position significantly supresses respiratory movements compared to whole-body PET/CT scans; therefore, even if the lesion is located at the edge of the detector, dbPET may show higher lesion visibility than PET/CT.

dbPET achieves higher geometric sensitivity and spatial resolution than whole-body PET/CT by (i) DOI detector and smaller voxel size in the former than in the latter, (ii) reduction of respiratory movement of the breast by acquisition in the prone position, and (iii) bringing the detector close to the breast. Especially, the 4-layer DOI detector used in dbPET can maintain geometric sensitivity and spatial resolution at the edges of the coronal field of view [28, 29]. The 4-layer DOI detector can detect gamma-ray interaction positions accurately by detecting Compton scattering events, and DOI information can dramatically reduce the voxel size. The voxel volume of dbPET (0.47 mm^3^) image was 178 times lower than that of PET/CT (84.5 mm^3^) image in this study. Additionally, when the background mammary gland showed physiological FDG uptake, the measured dbPET contrast was higher than the measured PET/CT contrast in the same lesion. As a result of earlier studies [30–32], the 2018 edition of the Japanese Guidelines for the Practice of Breast Cancer newly described the use of high-resolution breast PET as a supplemental modality for breasts with high density on mammography. Consequently, dbPET is expected to be applied to young women who often have high-density breasts. Both dbPET and PEM have the disadvantage that, due to their structural features, a part of the mammary gland near the chest wall is in a blind area and the lesion may be outside the field of view. However, this study demonstrates that if the lesion is within the field of view of dbPET, it can be detected with high probability, beyond 2 cm from the edge of the detector. Further studies are needed to classify in which patients and/or what lesions are likely to be located outside the FOV of either dbPET or PEM.

Because dbPET has much better performance characteristics, the lesions that can be detected by PET/CT would be more easily visualised. However, given the prognosis of breast cancer, comparison between both systems should focus on smaller lesions. The spatial resolution of whole-body PET/CT has improved due to the development of reconstruction techniques such as TOF and point spread function (PSF) modelling algorithms. In this study, we quantitatively evaluated TOF-reconstructed PET/CT images, since edge artefacts are known to occur in PSF modelling during reconstruction and are significant for small lesions [33]. Furthermore, some reports have shown that visualisation of small breast lesions can be improved by performing PET/CT in the prone position using assistive devices to allow breast expansion and suppression of respiratory movements [34, 35]. PET technologies, such as TOF and PSF, smaller pixel sizes, and prone position scanning, are expected to improve the visual detection rate of smaller lesions using PET/CT. This will allow a more direct future comparison of both devices for smaller lesions.

Our study had several limitations. First, the phantom was scanned only once for each position. The reproducibility of the findings would have been better if the average results of several scans under each condition were calculated. Second, the clinical study design was retrospective, and the patient cohort was small. Because only histologically proven breast cancers were included in this clinical study, small breast cancers near the edge of the detector that are false-negative on PET may not be sufficiently evaluated. Studies with larger populations and considerations including histology and subtypes of breast cancer will be required to address these limitations. Third, PET images acquired 90 min after injection are known to have improved uptake and contrast compared to those acquired 60 min after injection. Because this study was a retrospective study, all patients were scanned 60 and 90 min after FDG intravenous injection with PET/CT and dbPET, respectively, under our clinical conditions. This would likely have caused some bias in the results. Randomised prospective studies that appropriately control the start time of the scan are necessary for an accurate comparison of both devices.

## Conclusion

In our phantom study, image quality for all lesion sizes was worse when the phantom was within 2 cm of the edge of the detector. In the clinical studies, however, lesion visibility was the same regardless of whether the lesion was peripheral or non-peripheral, and the lesion visibility in both conditions was statistically significantly higher for dbPET than that in PET/CT.

## Data Availability

The datasets used and/or analysed during the current study are available from the corresponding author on reasonable request.

## Abbreviations

CT: Computed tomography
FOV: Field of view
ICC: Interclass correlation coefficients
PEM: Positron emission mammography
PET: Positron emission tomography/computed tomography
PSF: Point spread function
ROI: Region of interest
TBR: Tumour-to-background ratio
TOF: Time-of-flight
VOI: Volume of interest
